# The Effects of Xuefu Zhuyu and Shengmai on the Evolution of Syndromes and Inflammatory Markers in Patients with Unstable Angina Pectoris after Percutaneous Coronary Intervention: A Randomised Controlled Clinical Trial

**DOI:** 10.1155/2013/896467

**Published:** 2013-05-08

**Authors:** Jie Wang, Xiaochen Yang, Fuyong Chu, Jianxin Chen, Qingyong He, Kuiwu Yao, Fei Teng, Yonghong Gao, Yanhui Xing, Aiming Wu, Yanwei Xing

**Affiliations:** ^1^Guang'anmen Hospital, Chinese Academy of Chinese Medical Sciences, Xuan Wu, Beijing 100053, China; ^2^Beijing Hospital of Traditional Chinese Medicine, Dong Cheng, Beijing 100010, China; ^3^Beijing University of Chinese Medicine, Chao Yang, Beijing 100029, China; ^4^The Key Laboratory of Chinese Internal Medicine of the Ministry of Education, Dongzhimen Hospital Affiliated to Beijing University of Chinese Medicine, Dong Cheng, Beijing 100700, China; ^5^Institute of Information on Traditional Chinese Medicine, China Academy of Chinese Medical Sciences, Hai Dian, Beijing 100700, China

## Abstract

We evaluated the effects of the Xuefu Zhuyu capsule (XFZY) and the Shengmai capsule (SM) on the evolution of syndromes and inflammatory markers in patients with unstable angina pectoris (UAP) after percutaneous coronary intervention (PCI). Ninety patients with UAP after PCI were randomly and equally assigned to three groups: the XFZY group, the SM group, and the placebo group, with 30 patients in each group. Six syndrome factors (including Qi deficiency, yin deficiency, yang deficiency, blood stasis, phlegm, and Qi stagnation) and 4 inflammatory markers (high-sensitivity C-reactive protein (Hs-CRP), endothelins-1 (ET-1), matrix metalloproteinases-9 (MMP-9), and homocysteine (Hcy)) were observed at week 0 and at the 1st, 4th and 12th weeks. In conclusion, the evolution of syndromes present in patients with UAP after PCI followed these trends (1) The deficiency syndromes gradually increased during a 12-week period, but the excess syndromes first gradually decreased and then mildly increased after PCI. (2) XFZY and SM can prevent excess syndromes from increasing in the later stages and prevent deficiency syndromes from increasing in all stages. (3) XFZY and SMcan reduce the levels of the inflammatory markers, especially in the later stages after PCI.

## 1. Introduction

Coronary heart disease (CHD) is becoming the most common cause of death because infectious diseases have been controlled and living conditions have improved. In China, studies showed that the incidence of CHD increased by 26.1% for males and 19.0% for females from 1998 to 2008 [1]. In western countries, for instance, in the United States, an estimated 15,800,000 Americans have CHD, 7,900,000 have myocardial infarction (MI), and 8,900,000 have angina pectoris (AP) [2]. Unstable angina (UA) is more serious than stable AP, which can lead to heart attack and emergency medical care. Patients with acute MI or UA will be consistently treated with percutaneous coronary intervention (PCI) with stent placement. This standard nonsurgical treatment is effective in relieving the symptoms of coronary ischemia [3]. However, there are also some limitations for coronary stenting such as bare-metal stents, which may result in in-stent restenosis (ISR) due to the formation of scar tissues over the stent [4]. ISR doubles the risk of coronary ischemia and repeat revascularisation. Recently, with the development and the universal application of the drug eluting stent (DES), restenosis has been reduced to <10% for DESs [5]. However, several lines of evidence have showed that DESs do not reduce late cardiac-related death and the incidence of myocardial infarction (MI) because of stent thrombosis [6–9]. The treatment of ISR remains a challenging clinical issue.

Traditional Chinese medicine (TCM) has a 3000-year-old history that includes unique theories for aetiology and systems of diagnosis and treatment [10]. In TCM, the diagnosis, clinical evaluation, and treatment of CHD are based on signs and subjective symptoms according to the unique concept of “wholism.” In the Canon of Internal Medicine and Synopsis of Golden Chamber, CHD belongs to the classifications of the “obstruction of Qi in the chest” or “palpitation.” All CHD treatments are based on TCM diagnostics, and syndrome differentiation is essential. According to TCM, syndromes are the comprehensive responses of a certain stage in the disease process. The severity and prognosis of AP in patients with CHD are associated with the dynamic changes in a syndrome. Syndromes may significantly differ between patients with AP after PCI compared with before PCI. The syndrome factors refer to the major elements that comprise the syndromes. The main syndrome factors of AP include blood stasis, Qi deficiency, phlegm, yin deficiency, yang deficiency, and Qi stagnation. Comparison of the syndrome factors before and after PCI showed that the Qi deficiency and phlegm syndromes were progressively aggravated, whereas the Qi stagnation and cold coagulation syndromes were alleviated after PCI [11]. Therefore, it is important to elucidate the dynamic evolution of syndromes in patients with AP before and after PCI.

Recently, interest in traditional Chinese patent medicine (TCPM), which has been accepted by people for over 30 years, is rapidly increasing, especially its integration with routine western medical interventions. In China, at least one TCPM is used regularly in patients with AP after PCI in either western medicine hospitals or traditional Chinese medicine hospitals because it ameliorates ISR after PCI. Some researchers have reported the beneficial effect of TCPM on clinical symptoms, biomarkers, and mortality in patients with AP after PCI [12–14]. 

The Xuefu Zhuyu capsule (XFZY), a commonly used TCPM for removing blood stasis, has effectively treated patients with CHD [15]. The preparation of the Xuefu Zhuyu Decoction consists of 11 Chinese herbs: rehmannia root (shengdi), peach seed (taoren), Safflower (honghua), Chinese angelica (danggui), red peony root (chishao), platycodon root (jiegeng), orange fruit (zhiqiao), hare's ear root (chaihu), Sichuan lovage root (chuanxiong), two-toothed achyranthes root (niuxi), and prepared liquorice root (gancao). Xuefu Zhuyu has been a famous formula for treating blood stasis in the chest since the Qing dynasty. Xuefu Zhuyu has been pharmaceutically prepared into high-standard capsules. Experimental studies have shown that XFZY can increase coronary blood flow, improve cardiac microcirculation, prevent platelet aggregation, and accommodate blood lipids [16, 17]. In addition, the Sheng Mai capsule (SM), another commonly used TCPM for tonifying Qi, has effectively treated patients with CHD. It consists of 3 herbs: Radix Ophiopogonis (maidong), Fructus Schisandrae Chinensis (wuweizi), and Radix et Rhizoma Ginseng (renshen). However, the evidence supporting or disproving the clinical effects of TCPM on post-PCI UA patients is not robust. In this study, a randomised placebo-controlled clinical trial was conducted to observe the effects of XFZY and SM on the evolution of syndrome and inflammation markers of UAP before and after PCI.

## 2. Methods

### 2.1. Diagnostic Standard

The diagnostic criterion for UA was in reference to the American College of Cardiology and American Heart Association (ACC/AHA) 2002 Guidelines [18]. The blood stasis syndrome criteria were established by the Specialty Committee of Activating Blood and Removing Stasis, The Chinese Association of Integrative Medicine in 1986 [19]. The deficiency syndrome criteria were established by the Deficiency Syndrome and Geriatrics Society, the Chinese Association of Integrative Medicine in 1986 [20].

### 2.2. Inclusion and Exclusion Criteria

The inclusion criteria were participants who (1) were between 18 and 75 years old, (2) were diagnosed with UA following successful PCI therapy, and (3) signed the informed consent. The exclusion criteria were as follows: (1) patients with disorders that may affect the information collection process, such as senile dementia and psychosis; (2) patients with liver or kidney dysfunction; (3) patients that used other Chinese herbal medicines within two weeks; (4) patients with drug allergies; and (5) pregnant or lactating females.

### 2.3. Randomisation and Blinding

We applied the SAS 8.1 ProcPlan (Cary, NC, USA) to generate the random allocation sequences, and they were concealed in opaque envelopes beforehand. We entrusted the Clinical Pharmacological Research Centre of Guang'anmen Hospital to protect the envelopes. They were responsible for the randomisation but not involved in statistical analysis at the end of the trial. The XFZY and SM had unified packaging and were randomly ordered according to the random number table. During our trial, all clinical investigators, patients, and the statistical analyst were blinded to the distribution scheme. 

### 2.4. Participants and Treatment Process

We selected participants from March 2008 to February 2009. Ultimately, 90 patients with UA were enrolled from the Rescue Centre of Emergency, Anzhen Hospital, China Capital University of Medical Science. All of the patients who participated in this trial provided informed consent, and the protocol of this study was approved by the ethics committee of Guang'anmen Hospital and Anzhen Hospital. 

The 90 patients were randomly assigned to three groups: the XFZY group, SM group, and placebo group with 30 patients in each group. The patients in the XFZY group were given XFZY (Hong Ren Tang Pharmaceutical Co., Ltd., Tianjin, China, batch number: H03020) and the placebo of SM; the SM group was given SM (Chiatai Qing Chun Bao Pharmaceutical. Co., Ltd., Hangzhou, China, batch number: 0803001) and the placebo of XFZY; and the placebo group, simultaneously, received the placebos for XFZY and SM. Each medication and corresponding placebo were taken orally 30 min before meals, three capsules once and then three times daily from the second week after PCI for at least 12 weeks. The placebo, of which the colour, taste, and packaging were identical to the genuine agents, was made of starch and provided by the two respective pharmaceutical companies stated above. Aside from TCPM, the patients in each group were all given routine western therapy as their baseline therapy which included a 0.4–0.6 mL subcutaneous injection of enoxaparin every 12 hours for seven days, 100 mg aspirin and 75 mg clopidogrel once daily, 12.5–50 mg metoprolol tartrate once or twice daily according to heart rate, and 10 mg atorvastatin once daily. 

The study was conducted according to the guidelines of the Declaration of Helsinki and the principles of Good Clinical Practice (China) and obtained the approval of the medical ethics committee in Guang'anmen Hospital. The full trial protocol was drawn up and taken care by the Center for Drug Clinical Research of Guang'anmen Hospital, Chinese Academy of Chinese Medical Sciences.

### 2.5. Syndrome Factors

A syndrome factor is defined as the smallest taxon of pathogenesis for treating disease with TCM, which is the most basic unit of syndrome. It is associated with the physiological and pathological process of disease and can directly measure clinical information, which is related with the same pathogenesis. According to the provisions defined above, the syndrome factor should meet two conditions: first, the syndrome factor has a concept of pathogenesis, and second, it is quantifiable and cannot be abstract.

After extensive research in our research group, six major syndrome factors of AP were identified, blood stasis, Qi deficiency, phlegm, yin deficiency, yang deficiency, and Qi stagnation. Concurrently, we created a diagnostic scale of syndrome factors for angina pectoris (AP) (Table 2).

### 2.6. Outcome Measures

The patients were asked to visit the outpatient clinics at week 0, the 1st week, 4th week, and the 12th week. Electrocardiogram (ECG) and safety indices (blood routine and liver and kidney function tests) were examined at the beginning and after treatment (week 0, week 1, week 4, and week 12). Six syndrome factors (including Qi deficiency, yin deficiency, yang deficiency, blood stasis, phlegm, and Qi stagnation) and 4 inflammatory markers (Hs-CRP, ET-1, MMP-9, and Hcy) were observed at week 0, the 1st week, the 4th week, and 12th week. An ELISA assay was used to analyse Hs-CRP (R. B Corporation, USA), ET-1 (R. B Corporation, USA), MMP-9 (R. B Corporation, USA), and Hcy (R.B corporation, USA) levels.

### 2.7. Statistical Analysis

All of the experimental data were expressed as the means ± standard deviations (SDs). The data were statistically evaluated using one-way analysis of variance (ANOVA), and a post hoc test was performed using the least significant difference (LSD) (Figure 3 and Table 1). Fisher 2-sided exact tests are shown in Figure 2 and Table 1. The SPSS computer program, version 17.0, was used for the analyses. A probability of *P* < 0.05 was considered statistically significant.

## 3. Results

### 3.1. Baseline Characteristics of the Patients in the Different Groups

**Figure 1:**
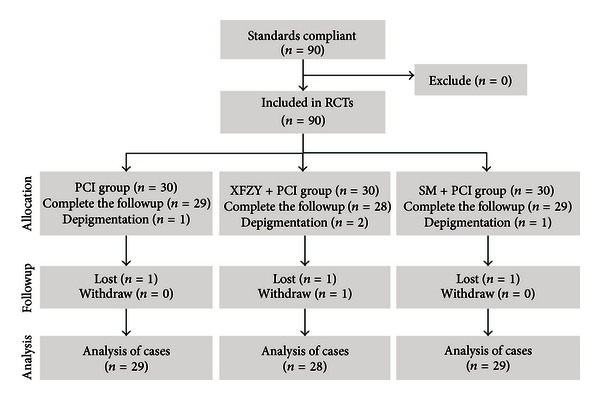
Participant recruitment, allocation, followup, and analysis.

In this study, there were four patients who withdrew from the trial; the attrition rate was 4.4% (4/90). One patient in the XFZY group withdrew from the study due to stomach discomfort after two weeks of the treatment, and another three patients from the XFZY, SM, and control groups withdrew due to their unwillingness to continue. Therefore, 86 patients completed the whole trial and were included in the final analysis. The baseline characteristics, including gender, age, disease duration, and concomitant condition, of the patients in the three groups were well matched (Table 1). The flow diagram of this randomised trial is shown in Figure 1.

### 3.2. The Distribution of Syndrome Factors in Unstable Angina Pectoris of Coronary Heart Disease

In this study, there were 90 patients enrolled who were diagnosed with unstable angina pectoris of coronary heart disease. Eighty-six patients completed the whole trial and were entered in the final analysis (Table 3). There were 6 syndrome factors that were closely related to UA, Qi deficiency (48%), yin deficiency (37%), yang deficiency (29%), blood stasis (69%), phlegm (48%), and Qi stagnation (41%). 

### 3.3. Effects of XFZY and SM on the Evolution of Syndrome Factors in the Patients with Unstable Angina Pectoris

**Figure 2:**
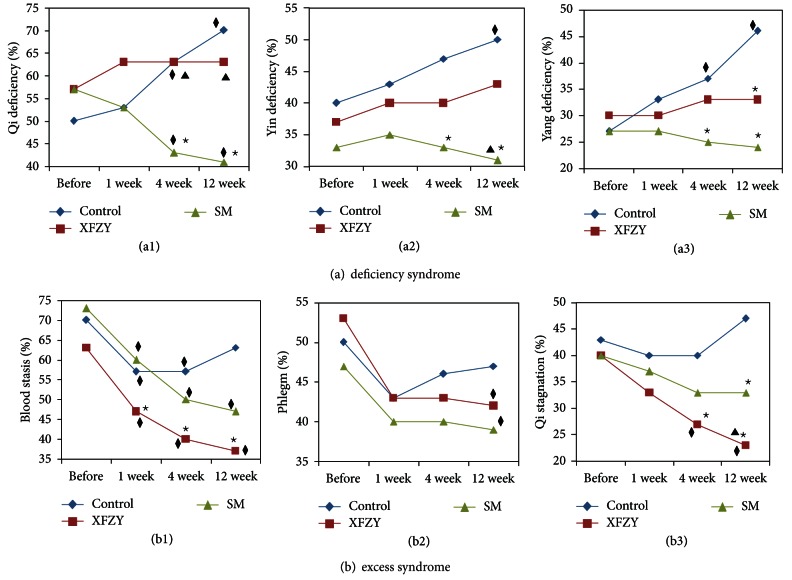
Effects of XFZY and SM on the evolution of syndrome factors in the patient with unstable angina pectoris. (a1) Effects of XFZY and SM on the Qi deficiency syndrome. (a2) Effects of XFZY and SM on the yin deficiency syndrome. (a3) Effects of XFZY and SM on the yang deficiency syndrome. (b1) Effects of XFZY and SM on the blood stasis syndrome. (b2) Effects of XFZY and SM on the phlegm syndrome. (b3) Effects of XFZY and SM on the Qi stagnation syndrome; (^★^ *P* < 0.05 versus the control group at the same time; ^◆^ *P* < 0.05 versus before treatment in the same group. ^▲^ *P* < 0.05, XFZY group versus SM group at the same time).

Compared with before the treatment, the Qi deficiency syndrome significantly increased by 14% (*P* < 0.05) and 21% (*P* < 0.05) by the 4th and 12th weeks, respectively, in the control group, significantly decreased by 13% (*P* < 0.05) and 16% (*P* < 0.05) by the 4th and 12th weeks, respectively, in the SM group, and did not significantly change in the XFZY group (*P* > 0.05) (Figure 2(a1)). Compared with the XFZY group, the Qi deficiency syndrome significantly decreased by 19% (*P* < 0.05) and 21% (*P* < 0.05) by the 4th and 12th weeks, respectively, in the SM group (Figure 2(a1)). Compared with the control group, the Qi deficiency syndrome significantly decreased by 20% (*P* < 0.05) and 30% (*P* < 0.05) by the 4th and 12th weeks, respectively, in the SM group (Figure 2(a1)).

Compared with before the treatment, the yin deficiency syndrome significantly increased by 13% by the 12th week in the control group (*P* < 0.05) (Figure 2(a2)). Compared with the control group, the yin deficiency syndrome significantly decreased by 17% (*P* < 0.05) and 19% (*P* < 0.05) by the 4th and 12th weeks, respectively, in the SM group (Figure 2(a2)).

Compared with before the treatment, the yang deficiency syndrome significantly increased by 10% (*P* < 0.05) and 19% (*P* < 0.05), respectively, by the 4th and 12th week in the control group (Figure 2(a3)). Compared with the control group, the yang deficiency syndrome significantly decreased by 11% (*P* < 0.05) and 19% (*P* < 0.05) by the 4th and 12th weeks, respectively, in the SM group (Figure 2(a3)). 

Compared with before the treatment, the blood stasis syndrome significantly decreased by 13% (*P* < 0.05) and 13% (*P* < 0.05) by the 1st and 4th weeks, respectively, in the control group, significantly decreased by 13% (*P* < 0.05), 22% (*P* < 0.05) and 25% (*P* < 0.05) by the 1st, 4th and 12th weeks, respectively, in the SM group and significantly decreased by 16%, (*P* < 0.05), 22% (*P* < 0.05) and 26% (*P* < 0.05) by the 1st, 4th and 12th weeks, respectively, in the SFZY group (Figure 2(b1)). Compared with the control group, the blood stasis syndrome significantly reduced by 13% (*P* < 0.05) and 13% (*P* < 0.05) by the 1st and the 4th weeks, respectively, significantly decreased by 16% (*P* < 0.05) and 27% (*P* < 0.05) by the 1st, 4th and 12th weeks, respectively, in the XFZY group and significantly decreased by 16% (*P* < 0.05) by the 12th week in the SM group (Figure 2(b1)). Compared with the SM group, the blood stasis syndrome significantly decreased by 13% (*P* < 0.05), 10% (*P* < 0.05) and 11% (*P* < 0.05) by the 1st, 4th and 12th weeks, respectively, in the XFZY group (Figure 2(b1)).

Compared with before the treatment, the phlegm syndrome significantly decreased by 10% (*P* < 0.05) by the 12th week in the SM group (Figure 2(b2)). In the XFZY group, the phlegm syndrome significantly decreased by 12% by the 12th week (*P* < 0.05). 

Compared with before the treatment, the Qi stagnation syndrome significantly decreased by 13% (*P* < 0.05) and 17% (*P* < 0.05) by the 4th and 12th weeks, respectively, in the XFZY group (Figure 2(b3)). Compared with the control group, the Qi stagnation syndrome significantly decreased by 13% (*P* < 0.05) and 23% (*P* < 0.05) by the 4th and 12th weeks, respectively, in the XFZY group. Compared with the SM group, the Qi stagnation syndrome significantly decreased by 12% (*P* < 0.05) by the 12th weeks in the XFZY group.

### 3.4. Effects of XFZY and SM on Hs-CRP, ET-1, MMP-9 and Hcy

**Figure 3:**
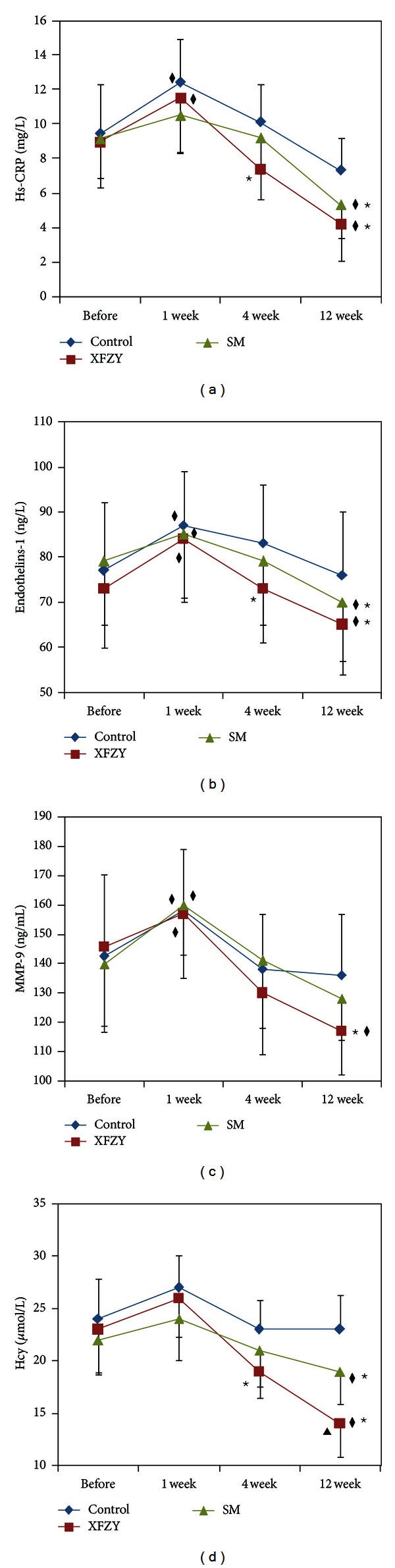
Effects of XFZY and SM on Hs-CRP, ET-1, MMP-9 and Hcy. (a) Effects of XFZY and SM on Hs-CRP. (b) Effects of XFZY and SM on ET-1. (c) Effects of XFZY and SM on MMP-9. (d) Effects of XFZY and SM on Hcy; (^★^ *P* < 0.05 versus the control group at the same time; ^◆^ *P* < 0.05 versus before treatment in the same group; ^▲^ *P* < 0.05, XFZY group versus SM group at the same time).

Compared with before the treatment, Hs-CRP significantly increased by the 1st week in the XFZY group and control group (*P* < 0.05) but significantly decreased by the 4th and 12th weeks in the XFZY group Hs-CRP (*P* < 0.05), and significantly decreased by the 12th week in the SM group Hs-CRP (*P* < 0.05) (Figure 3(a)). Compared with the control group, Hs-CRP significantly decreased by the 4th and 12th weeks in the XFZY group (*P* < 0.05) and significantly decreased by the 12th week in the SM group (*P* < 0.05) (Figure 3(a)).

Compared with before the treatment, ET-1 significantly increased by the 1st week in the control group, XFZY group and SM group (*P* < 0.05) but significantly decreased by the 4th and 12th weeks in the XFZY group (*P* < 0.05) and significantly decreased by the 12th week in the SM group (*P* < 0.05) (Figure 3(b)). Compared with the control group, ET-1 significantly decreased by the 4th and the 12th weeks in the XFZY group (*P* < 0.05) and significantly decreased by the 12th week in the SM group (*P* < 0.05) (Figure 3(b)).

Compared with before the treatment, MMP-9 significantly increased by the 1st week in the control group, XFZY group and SM group (*P* < 0.05) but significantly decreased by the 12th weeks in the XFZY group (*P* < 0.05) (Figure 3(c)). Compared with the control group, MMP-9 significantly decreased by the 12th weeks in the XFZY group (*P* < 0.05).

Compared with before the treatment, Hcy significantly decreased by the 12th weeks in the XFZY group (*P* < 0.05) (Figure 3(d)). Compared with the control group, Hcy significantly decreased by the 4th and 12th weeks in the XFZY group, (*P* < 0.05) and significantly decreased by the 12th week in the SM group (*P* < 0.05) (Figure 3(d)). Compared with the SM group, Hcy significantly decreased by the 12th weeks in the XFZY group (*P* < 0.05).

## 4. Discussion

We can draw several conclusions from the present study: (1) The evolution of syndromes in patients with UAP after PCI occurred with the following trends: the deficiency syndromes including Qi deficiency, yin deficiency and yang deficiency gradually increased until 12 weeks, but the excess syndromes including blood stasis, phlegm and Qi stagnation first gradually decreased and then mildly increased until week 12. (2) XFZY and SM can prevent excess syndromes from increasing in the later stages and prevent deficiency syndromes from increasing during all stages. (3) XFZY and SM can prevent inflammatory markers (Hs-CRP, ET-1, MMP-9, and Hcy) from increasing, especially in the later stages. 

### 4.1. Interestingly, the Deficiency Syndromes Gradually Increased during All Stages, but the Excess Syndromes First Gradually Decreased and Then Mildly Increased Later

Although PCI therapy can rapidly relieve myocardial ischemia and reduce mortality due to acute myocardial infarction, PCI therapy has been found to only focus on the local coronary lesions but does not affect the whole body function recovery. In traditional Chinese medical theory, PCI can be attributed to the “eliminating evil” therapy with an effect on “activating” blood circulation to remove stasis. due to the “broken blood” effect of PCI, genuine Qi is gradually damaged so that the deficiency syndromes are prioritised in patients after PCI. The Qi deficiency and increase in evil must be combined, with the Qi disorder, blood running, blood stasis, and phlegm endogenous; the context is again blocked by blood stasis, which ultimately induces chest pain [21]. Therefore, after PCI treatment, the patients should mainly prioritise tonifying Qi and lifting yang while complementarily eliminating pathogenic therapy.

In this study, the deficiency syndromes gradually increased, and the excess syndromes (Qi stagnation, blood stasis and phlegm turbidity) initially decreased and then gradually increased postoperatively. The syndrome changes increased in complexity, but we found that the late Qi deficiency and blood stasis after PCI still represented the essence of disease pathogenesis.

### 4.2. XFZY and SM Can Prevent Excess Syndromes from Increasing during the Later Stages and Prevent Deficiency Syndromes from Increasing during All Stages

In this study, we found that the deficiency syndromes increased gradually after PCI, the excess syndromes initially decreased and then increased and the Qi deficiency and blood stasis were still the main pathogenesis of the disease. SM and XFZY are classical prescriptions for tonifying Qi and promoting blood circulation, and their curative effects are distinct in the treatment of coronary heart disease. This study found that XFZY and SM could prevent excess syndromes from increasing within 4 weeks after PCI and prevent the deficiency syndromes from increasing during all stages; the syndromes became more simplified. Although SM is a topical prescription for tonifying Qi that can also improve blood stasis, XFZY is a treatment for Qi stagnation and blood stasis that can also improve Qi deficiency syndromes after PCI.

### 4.3. XFZY and SM Can Prevent Inflammatory Markers (Hs-CRP, ET-1, MMP-9, and Hcy) from Increasing, Especially during the Later Stages

Hs-CRP, a plasma inflammation marker, has been known to play a role in the development of cardiovascular diseases and is considered as a biomarker that predicts early cardiovascular risk [22]. Several prospective epidemiological studies have demonstrated a consistent relationship between higher C-reactive protein (CRP) levels and an increased risk of cardiovascular events, including myocardial infarction, stroke, and cardiovascular death [23, 24]. CRP may reflect a greater burden of atherosclerosis or alternatively may identify a high-risk atherosclerosis phenotype with an active inflammation and atherosclerotic plaque that is vulnerable to rupture [25, 26]. In this study, we found that Hs-CRP increased by the 1st week in all groups, but compared with the control group and before the treatment, Hs-CRP significantly decreased later in the XFZY group, and SM group. We hypothesised that PCI-induced Hs-CRP level increases by the 1st week. XFZY and SM could decrease CRP and cardiovascular events. 

The current study extends these findings by demonstrating that depression symptom severity is related to the resting level of ET-1, a protein involved in the regulation of vascular compliance, and is directly linked to plaque rupture [27, 28]. Each point increase in depression severity independently increased the likelihood of a patient with a resting level of ET-1 in a range previously found to predict post-ACS morbidity and mortality by 14% [29, 30]. The likelihood increased to 3.75-fold when a threshold of depressive symptoms that has repeatedly been linked to post-ACS prognosis was achieved. We found that ET-1 levels increased by the 1st week in all of the groups, but compared with the control group and before the treatment, ET-1 levels significantly decreased later in the XFZY group and SM group. Compared with the SM group, the ET-1 levels significantly decreased by the 12th weeks in the XFZY group. XFZY and SM could decrease ET-1 levels and prevent inflammatory response.

MMP-9 is associated with atherosclerotic arterial remodelling [31] and is actively synthesised in vulnerable plaques [32]. MMP-9 affects plaque stability in association with various inflammatory cytokines [33, 34]. Elevated MMP-9 plasma levels in the peripheral blood have been detected in the patients with ACS [35] and are associated with severe coronary stenosis [36] and cardiovascular mortality [37]. In addition, the level of plasma MMP-9 is elevated in the coronary circulation of patients with ACS [38, 39], indicating that the production of MMP-9 may be enhanced in ACS. We found that MMP-9 levels increased by the 1st week in all of the groups, but compared with the control group and before the treatment, MMP-9 levels significantly decreased later in the XFZY and SM groups. XFZY and SM could decrease the MMP-9 level to prevent the inflammatory response.

Hcy is a well-known mixed amino acid intermediary between methionine and cysteine in the metabolic pathway [40]. Mild to moderate elevation of the plasma, Hcy concentration has been known to increase the risk for developing atherosclerotic vascular disease [41]. Hyperhomocysteinemia is an independent risk factor for atherosclerosis, and impaired arterial endothelial function is detectable in healthy adults with hyperhomocysteinemia [41]. An elevated homocysteine level was recently identified as an independent risk factor for coronary artery disease and premature atherosclerosis. Compared with the control group and before the treatment, we found that the level of homocysteine increased by the 1st week in all of the groups but later significantly decreased in the XFZY and SM groups (Figure 3(a)). Compared with the SM group, Hcy levels significantly decreased by the 12th weeks in the XFZY group. XFZY and SM could decrease Hcy levels and prevent inflammatory response.

## 5. Conclusion

The evolution of syndromes in patients with UAP after PCI occurred with the following trends: the deficiency syndromes including Qi deficiency, yin deficiency, and yang deficiency gradually increased in all of the stages, but the excess syndromes including blood stasis, phlegm and Qi stagnation initially gradually decreased and then mildly increased. XFZY and SM can prevent excess syndromes from increasing in the later stages and prevent the deficiency syndromes from increasing in all stages. XFZY and SM can prevent inflammatory markers (Hs-CRP, ET-1, MMP-9, and Hcy) from increasing, especially during the later stages.

## Figures and Tables

**Table 1:** Baseline characteristics of the patients in the different groups.

Items	Control group (30 cases)	XFZY group (30 cases)	SM group (30 cases)	Statistical value	*P* value
Males (case (%))	20 (66.7)	18 (60.0)	15 (50.0)	1.96	0.42
Age (year)	58.8 ± 8.9	61.7 ± 9.6	61.6 ± 9.2	0.14	0.87
Course (year)	6.1 ± 3.4	5.2 ± 3.2	5.8 ± 3.7	0.22	0.65
Body mass index (Kg/M^2^)	23.28 ± 3.59	23.40 ± 3.54	24.07 ± 3.41	0.12	0.89
Hypertension (case (%))	12 (40.0)	13 (43.3)	14 (46.7)	0.31	0.87
Hyperlipidaemia (case (%))	10 (33.3)	11 (36.7)	10 (33.3)	0.19	0.95
Diabetes mellitus (case (%))	12 (40.0)	7 (23.3)	11 (36.7)	1.87	0.35
Smoking (case (%))	19 (63.3)	17 (56.7)	15 (50.0)	1.18	0.56
No. of planted stents	1.7 ± 1.2	1.9 ± 1.1	1.2 ± 0.8	1.21	0.31

**Table 2:** Diagnostic scale of syndrome factors for AP.

Syndrome factor	Major symptoms	Minor symptoms
Qi deficiency	Spontaneous sweat; fatigue induced by labour; fat tongue with tooth marks	Dizziness; palpitations
Yin deficiency	Faint chest pain; night sweats; five upset hot; red tongue with less moss	Dry mouth; dry eyes; insomnia; palpitations
Yang deficiency	Chest tightness and pain; aversion to cold; abdominal and waist cold; pale and fat tongue	Nocturia; face and paw edema; lassitude
Blood stasis	Chest pain; petechia and ecchymosis; dark complexion; dark or purple tongue	Squamous and dry skin; numbness of limbs; dark red lip
Phlegm	Chest pain; abdominal distention; sticky mouth; nausea; thick and greasy moss	Fat body; dizziness and more sleep; fatigue; shortness of breath
Qi stagnation	Chest tightness and pain; distending pain in hypochondrium; irritability	Depression; distension and fullness belching

Diagnostic criteria of syndrome factors include no less than the two major symptoms and one minor symptom.

**Table 3:** Frequency distribution of syndrome factors in the different groups.

Syndrome factors	Before treatment (case (%))			1st week (case (%))			4th week (case (%))			12th week (case (%))		
	Control	XFZY	SM	Control	XFZY	SM	Control	XFZY	SM	Control	XFZY	SM
Qi deficiency (case (%))	15 (50)	16 (57)	17 (57)	16 (53)	19 (63)	16 (53)	18 (64)	19 (63)	13 (44)	20 (71)	18 (62)	12 (41)
Blood stasis (case (%))	21 (70)	19 (63)	22 (73)	17 (57)	14 (47)	18 (60)	16 (57)	12 (41)	15 (51)	18 (64)	11 (37)	14 (48)
Yin deficiency (case (%))	12 (40)	11 (37)	10 (33)	13 (43)	12 (40)	11 (37)	14 (50)	12 (41)	10 (33)	16 (53)	13 (43)	10 (34)
Phlegm (case (%))	15 (50)	16 (53)	12 (40)	13 (43)	13 (43)	12 (40)	13 (46)	13 (44)	12 (41)	13 (46)	12 (41)	11 (38)
Qi stagnation (case (%))	13 (43)	12 (40)	12 (40)	12 (40)	10 (33)	11 (37)	11 (40)	8 (27)	9 (33)	13 (46)	7 (23)	10 (34)
Yang deficiency (case (%))	9 (27)	9 (30)	8 (27)	10 (33)	9 (30)	8 (27)	11 (37)	10 (33)	9 (26)	13 (46)	10 (34)	8 (27)

## References

[1] Yangfeng W (2001). Current status of major cardiovascular risk factors in Chinese populations and their trends in the past two decades. *Chinese Journal of Cardiology*.

[2] Rosamond W, Flegal K, Furie K (2008). Heart disease and stroke statistics-2008 update: a report from the American Heart Association statistics committee and stroke statistics subcommittee. *Circulation*.

[3] Grech ED (2003). ABC of interventional cardiology: percutaneous coronary intervention. II: the procedure. *British Medical Journal*.

[4] Ma X, Wu T, Robich MP (,2010). Drug-eluting stents. *International Journal of Clinical and Experimental Medicine*.

[5] Aziz S, Morris JL, Perry RA (2007). Late stent thrombosis associated with coronary aneurysm formation after sirolimus-eluting stent implantation. *Journal of Invasive Cardiology*.

[6] Kim HL, Park KW, Kwak JJ (2008). Stent-related cardiac events after non-cardiac surgery: drug-eluting stent versus bare metal stent. *International Journal of Cardiology*.

[7] Camenzind E (2006). Treatment of in-stent restenosis—back to the future?. *New England Journal of Medicine*.

[8] Pfisterer ME, Richartz B, Silber S (2006). The BASKET-LATE study: basel stent cost-effectiveness trial—late thrombotic events trial. *Herz*.

[9] Ten Berg JM, Thijs Plokker HW, Verheugt FWA (2001). Antiplatelet and anticoagulant therapy in elective percutaneous coronary intervention. *Current Controlled Trials in Cardiovascular Medicine*.

[10] Keji C, Hao X (2003). The integration of traditional Chinese medicine and Western medicine. *European Review*.

[11] Chen BJ, Pan ZQ, Su XX (2007). Study on changes of TCM syndrome in patients with coronary heart disease before and after intervention treatment. *Zhongguo Zhong Xi Yi Jie He Za Zhi*.

[12] Zheng GL, Wang SH (2009). Clinical effect and mechanismn of xuefu zhuyu capsule in treating unstable angina pectoris. *Zhongguo Zhong Xi Yi Jie He Za Zhi*.

[13] Wang XY, Lou Y (2009). Combination of XuefuZhuyu decoction with western medicine for unstable angina in 56 cases with coronary heart disease. *Tianjin Journal of Traditional Chinese Medicine*.

[14] Cai FS (2009). Observation on clinical therapeutic effect of Xuefuzhuyu Capsules in treating angina pectoris. *Journal of Liaoning University of Traditional Chinese Medicine*.

[15] Li YR, Zhang HF, Du J, SU DW (2008). Clinical observation of XuefuZhuyu Capsule for unstable angina in patients with coronary heart disease. *Journal of Liaoning University of Traditional Chinese Medicine*.

[16] Song J, Chen WY, Wu LY (2012). A microarray analysis of angiogenesis modulation effect of Xuefu Zhuyu Decoction on endothelial cells. *Chinese Journal of Integrative Medicine*.

[17] Zhang QY, Wang QL, Su JF (2011). Angiogenesis effects of Xuefu Zhuyu Decoction and VEGF protein expression of rats with acute myocardial Ischemia. *Chinese Journal of Information on Traditional Chinese Medicine*.

[18] Braunwald E, Antman EM, Beasley JW (2000). ACC/AHA guidelines for the management of patients with unstable angina and non-ST-segment elevation myocardial infarction: executive summary and recommendations. A report of the American College of Cardiology/American Heart Association task force on practice guidelines (committee on the management of patients with unstable angina). *Circulation*.

[19] Specialty Committee of Activating Blood and Removing Stasis CAOIM (1987). Diagnostic standard of blood stasis syndrome. *Chinese Journal of Integrated Traditional and Western Medicine*.

[20] Shen Z, Wang W (1986). Standards reference of TCM deficiency syndrome. *Chinese Journal of Integrated Traditional and Western Medicine*.

[21] Zhang MZ, Wang L (2006). The academic ideology of DENG TieTao treatment of sydroms after PCI based on syndrome differentiation. *Journal of Traditional Chinese Medicine*.

[22] Shroff GR, Cen YY, Duprez DA, Bart BA (2009). Relationship between carotid artery stiffness index, BNP and high-sensitivity CRP. *Journal of Human Hypertension*.

[23] Rost NS, Wolf PA, Kase CS (2001). Plasma concentration of C-reactive protein and risk of ischemic stroke and transient ischemic attack: the Framingham Study. *Stroke*.

[24] Koenig W, Sund M, Fröhlich M (1999). C-reactive protein, a sensitive marker of inflammation, predicts future risk of coronary heart disease in initially healthy middle-aged men: results from the MONICA (monitoring trends and determinants in cardiovascular disease) Augsburg cohort study, 1984 to 1992. *Circulation*.

[25] Verma S, Wang CH, Li SH (2002). A self-fulfilling prophecy: C-reactive protein attenuates nitric oxide production and inhibits angiogenesis. *Circulation*.

[26] Falk E, Shah PK, Fuster V (1995). Coronary plaque disruption. *Circulation*.

[27] Zeiher AM, Goebel H, Schachinger V, Ihling C (1995). Tissue endothelin-1 immunoreactivity in the active coronary atherosclerotic plaque: a clue to the mechanism of increased vasoreactivity of the culprit lesion in unstable angina. *Circulation*.

[28] Zhang X, Zhao F, Xu C (2008). Circadian rhythm disorder of thrombosis and thrombolysis-related gene expression in apolipoprotein E knock-out mice. *International Journal of Molecular Medicine*.

[29] Katayama T, Yano K, Nakashima H (2005). Clinical significance of acute-phase endothelin-1 in acute myocardial infarction patients treated with direct coronary angioplasty. *Circulation Journal*.

[30] Yip HK, Wu CJ, Chang HW (2005). Prognostic value of circulating levels of endothelin-1 in patients after acute myocardial infarction undergoing primary coronary angioplasty. *Chest*.

[31] Pasterkamp G, Schoneveld AH, Hijnen DJ (2000). Atherosclerotic arterial remodeling and the localization of macrophages and matrix metalloproteases 1, 2 and 9 in the human coronary artery. *Atherosclerosis*.

[32] Brown DL, Hibbs MS, Kearney M, Loushin C, Isner JM (1995). Identification of 92-kD gelatinase in human coronary atherosclerotic lesions: association of active enzyme synthesis with unstable angina. *Circulation*.

[33] Sarén P, Welgus HG, Kovanen PT (1996). TNF-*α* and IL-1*β* selectively induce expression of 92-kDa gelatinase by human macrophages. *Journal of Immunology*.

[34] Kim SH, Kang YJ, Kim WJ (2004). TWEAK can induce pro-inflammatory cytokines and matrix metalloproteinase-9 in macrophages. *Circulation Journal*.

[35] Kai H, Ikeda H, Yasukawa H (1998). Peripheral blood levels of matrix metalloproteases-2 and -9 are elevated in patients with acute coronary syndromes. *Journal of the American College of Cardiology*.

[36] Kalela A, Koivu TA, Sisto T (2002). Serum matrix metalloproteinase-9 concentration in angiographically assessed coronary artery disease. *Scandinavian Journal of Clinical and Laboratory Investigation*.

[37] Blankenberg S, Rupprecht HJ, Poirier O (2003). Plasma concentrations and genetic variation of matrix metalloproteinase 9 and prognosis of patients with cardiovascular disease. *Circulation*.

[38] Inokubo Y, Hanada H, Ishizaka H, Fukushi T, Kamada T, Okumura K (2001). Plasma levels of matrix metalloproteinase-9 and tissue inhibitor of metalloproteinase-1 are increased in the coronary circulation in patients with acute coronary syndrome. *American Heart Journal*.

[39] Funayama H, Ishikawa SE, Kubo N (2004). Increases in interleukin-6 and matrix metalloproteinase-9 in the infarct-related coronary artery of acute myocardial infarction. *Circulation Journal*.

[40] Aksungar FB, Topkaya AE, Akyildiz M (2007). Interleukin-6, C-reactive protein and biochemical parameters during prolonged intermittent fasting. *Annals of Nutrition and Metabolism*.

[41] Graham LM, Daly LE, Refsum HM (1997). Plasma homocysteine as a risk factor for vascular disease: the European Concerted Action Project. *Journal of the American Medical Association*.

